# Expression breadth and expression abundance behave differently in correlations with evolutionary rates

**DOI:** 10.1186/1471-2148-10-241

**Published:** 2010-08-07

**Authors:** Seung Gu Park, Sun Shim Choi

**Affiliations:** 1 Department of Medical Biotechnology, College of Biomedical Science, and Institute of Bioscience & Biotechnology, Kangwon National University, Chunchon 200-701, Korea

## Abstract

**Background:**

One of the main objectives of the molecular evolution and evolutionary systems biology field is to reveal the underlying principles that dictate protein evolutionary rates. Several studies argue that expression abundance is the most critical component in determining the rate of evolution, especially in unicellular organisms. However, the expression breadth also needs to be considered for multicellular organisms.

**Results:**

In the present paper, we analyzed the relationship between the two expression variables and rates using two different genome-scale expression datasets, microarrays and ESTs. A significant positive correlation between the expression abundance (EA) and expression breadth (EB) was revealed by Kendall's rank correlation tests. A novel random shuffling approach was applied for EA and EB to compare the correlation coefficients obtained from real data sets to those estimated based on random chance. A novel method called a Fixed Group Analysis (FGA) was designed and applied to investigate the correlations between expression variables and rates when one of the two expression variables was evenly fixed.

**Conclusions:**

In conclusion, all of these analyses and tests consistently showed that the breadth rather than the abundance of gene expression is tightly linked with the evolutionary rate in multicellular organisms.

## Background

Proteins in a species evolve at different rates [1]. The systems evolutionary genomics field studies the factors that determine the evolutionary rates of proteins. Over the last thirty years, since the neutral evolutionary theory was first suggested, a lack of sequence data prevented thorough investigation of protein evolution. One accepted consensus is that protein evolutionary rates are controlled by the density of amino acid residues in a protein under the influence of different functional constraints [2]. In other words, the functional importance of amino acid residues and their densities in a protein determine its evolutionary rate. This 'function-centered' hypothesis predicts several evolutionary outcomes. For example, proteins with high dispensability and a high propensity of gene loss (PGL) are expected to evolve more rapidly [3], whereas essential proteins and those at hub positions in a protein-protein interaction (PPI) network are predicted to evolve more slowly [4-6]. These hypotheses have been proven or disproven by various research groups through analyses of different data sets [3,7,8]. Recently, several research groups have investigated this issue using genome-scale data of sequences, mutants, and PPIs, and have concluded that some genomic parameters exhibit weak but statistically significant correlations with evolutionary rates [9-12].

Among the genomic parameters, expression level is the most prominent and consistent negative correlate with protein evolutionary rate in unicellular organisms [12-15]. About 20-40% of variation in protein evolutionary rates can be explained by the expression abundance. Drummond et al. (2008) argued that about half of the variation can be explained by the expression level [16]. Other correlates related to expression level lead to qualitatively similar results [17,18]. For example, in yeast, the divergence among paralogs after duplication is related to expression levels [19]. Principal component analyses also confirmed that protein abundance has a greater effect than any other variables in determining rates [12]. Moreover, the effect of dispensability and PPIs on rates diminishes when the expression abundance is controlled [16,20,21].

In expression-based evolutionary analysis, the estimation of expression abundance in multicellular organisms is more complicated than for unicellular organisms. Genes express at different levels in different tissue types in multicellular organisms. For instance, some genes express at high levels in specific tissue types while others are evenly expressed at low levels in all tissue types, indicating that broadly expressed genes are not necessarily highly expressed genes. It has been reported that ubiquitously expressed genes evolve more slowly than tissue-specific genes, which suggests that the extent to which genes express is critical for their evolutionary rates in multicellular organisms [18,22-24]. Accordingly, it remains unclear if the expression abundance is truly the most important correlate with evolutionary rates in multicellular organisms.

In this paper, we compared two different expression measures, namely expression abundance (EA) and expression breadth (EB), on their correlation with evolutionary rates using both microarrays and EST datasets. Our study may contribute to a better understanding of what determines the evolutionary rates of proteins in multicellular organisms.

## Results and Discussion

### Preparation of gene expression data from two different sources

**Figure 1:**
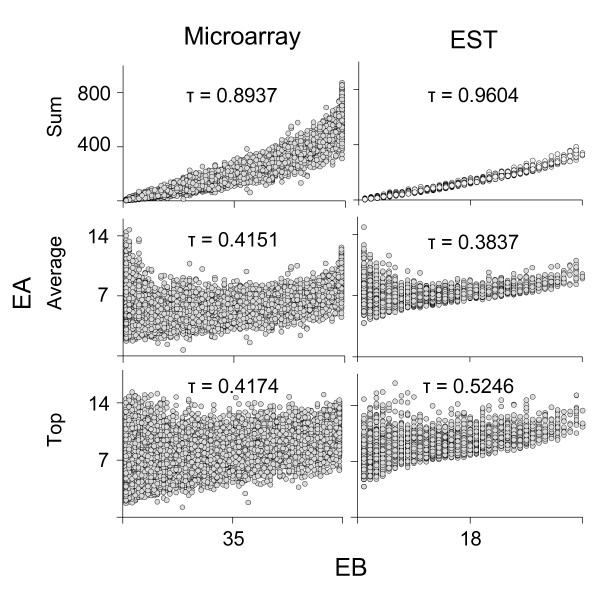
**Positive correlations between EBs and EAs in the two datasets from the ESTs and microarray**. Three different definitions of EA were used to investigate the correlation between EAs and EBs in the two data sets from the microarray (left) and ESTs (right). Detailed definitions of the three different values on the y-axis are described in the main text. Kendall's tau values are indicated in the upper middle section in each box plot. All graphs consistently show that EA is positively correlated with EB at statistical significance (p < 0.01), although the size of tau varies with different tests.

To investigate the relationship between expression parameters (EB and EA) and evolutionary rates, validated genome-wide expression datasets were needed. Two different expression datasets, GDS596 microarray data derived from the Gene Expression Omnibus (GEO) human database and EST data obtained from the UniGene database, were used in the present analysis (see Methods, Figure 1). Evolutionary rates such as *Ka*, *Ks*, and *Ka/Ks *were estimated based on orthologous pairs between human and mouse genes (Methods).

A previous report stated that the two data types exhibit an intrinsic difference in gene expression profiling [25]. According to Zhu et al. (2008), EST data are not saturated, so there is limited gene detectability for tissue-specific genes; in contrast, microarray data exhibit a higher false negative rate compared to EST data, leading to a significant underestimation of housekeeping genes [26]. We noticed various problems with the two datasets. For example, the greatest challenge in the analysis of microarray data is how to determine the cutoff for absent/present (AP) calls, while in EST data there is a big difference in the sizes of the cDNA libraries from different tissue types, ranging from hundreds to millions depending on the tissue. Microarray data present values of gene expression levels that are referred to as signal intensities, while ESTs determine the numbers of ESTs that are believed to indicate the level of gene expression. To overcome the limitations of the two databases, we applied several different cutoffs to estimate AP calls in the EST and microarray data (Methods). Strong positive correlations between different datasets generated by different methods suggested that the datasets are qualitatively similar (Additional file 1, Figure S1). In fact, the datasets generated from the different methods led to essentially the same conclusions as those in the present paper (data not shown).

Specific details including data cleaning and cutoffs are described in the Methods section. In summary, a total of 9,506 genes were chosen from 69 different adult normal tissue types after removing cancer, tumor, and fetal tissues from the microarray data, and a total of 13,605 genes consisting of 507,140 ESTs were selected from 36 different tissues. For these two data sets, all analyses were applied in parallel, and similar conclusions were reached from both. However, the results derived from the analysis of the microarray datasets will mostly be discussed in the present paper along with additional files from some of the analyses of EST datasets. A combination of the analyses from both datasets is expected to reduce the possibility of data misinterpretation.

### The positive correlation between EB and EA

The estimation of EB is relatively simple and is defined as the sum of the number of tissue types in which a given gene is expressed at or above a threshold value. Liao et al. (2006) used the τ value to measure the tissue specificity of gene expression [24], which is the inverse of expression broadness. We found that the conclusions generated by the two measures are not different overall, although the details may not be the same (data not shown). To estimate EA, two different estimations are applied in studies of gene expressions of multicellular organisms: (1) the average signal intensity (or average of a proportion of ESTs) of a specific gene expressed in a number of tissue types [16,27] or (2) the sum of signal intensities (or sum of a proportion of ESTs) of a specific gene expressed in a number of tissue types [28]. For unicellular organisms, measuring the 'abundance' involves determining the total transcripts of a specific gene expressed in the whole organism. For multicellular organisms, the estimation of 'abundance' by (2), the 'sum'-based estimation method, seems to be more reasonable in expression profiling studies, although more studies of gene expression are based on (1), the 'average'-based estimation method. The average-based estimation process has been used to remove the effect of broadness from abundance in the context of gene expression.

By using the definition within (2), EAs are essentially positively correlated with EBs. However, even when the definition within (1) is used, EA is positively correlated with EB [29]. Using either definition, EAs are positively correlated with EBs [12,15,19]. However, it is necessary to discriminate between how the two values influence the correlations with the evolutionary rates in order to better understand evolutionary mechanisms in multicellular organisms. Even when the average definition is used for EA, we reasoned that it is incorrect to say that a given gene expresses weakly when it actually expresses at a high level in a certain tissue type and at a low level in other tissue types. Therefore, we designed the third definition of EA, namely the highest signal intensity value (the TOP value) among the intensity values derived from all different tissues for a given gene as revealed by microarray data or the highest proportion value given by EST data.

Using the three different definitions of EA, we plotted EB against EA to see how strongly the breadth and abundance of gene expression are correlated. Interestingly, regardless of the definitions, broadly expressed genes are consistently more likely to express at high levels in both the microarray and EST datasets (Figure 1). The data points showed a more scattered pattern for microarrays than for the EST data; however, this difference was likely caused by the smaller number of tissue types represented in the EST data. The microarray data contained gene expression information for 69 different tissues, while the ESTs only had data for 36 different tissues. It is important to note that all analyses in the present paper used the three different definitions of EA in parallel and yielded essentially the same patterns; however, we will present the results mainly from the third definition of EA.

### Significant negative correlations between the expression variables, EA and EB, and evolutionary rates

Before we analyzed the correlation between expression parameters and evolutionary rates, we first investigated the relationship between expression parameters and expression divergence. Previously, the expression breadth divergence between two different species estimated by the expression conservation index (ECI) was reported to be positively correlated with the broadness of gene expression, meaning that broadly expressed genes are more likely to have conserved expression breadth [18]. The relationship between the expression level divergence between two different species was also studied. Genes with higher expression level divergence are more likely to be expressed at low levels [30]. We confirmed all of these conclusions (Additional file 1, Figure S2A and S2B).

**Figure 2:**
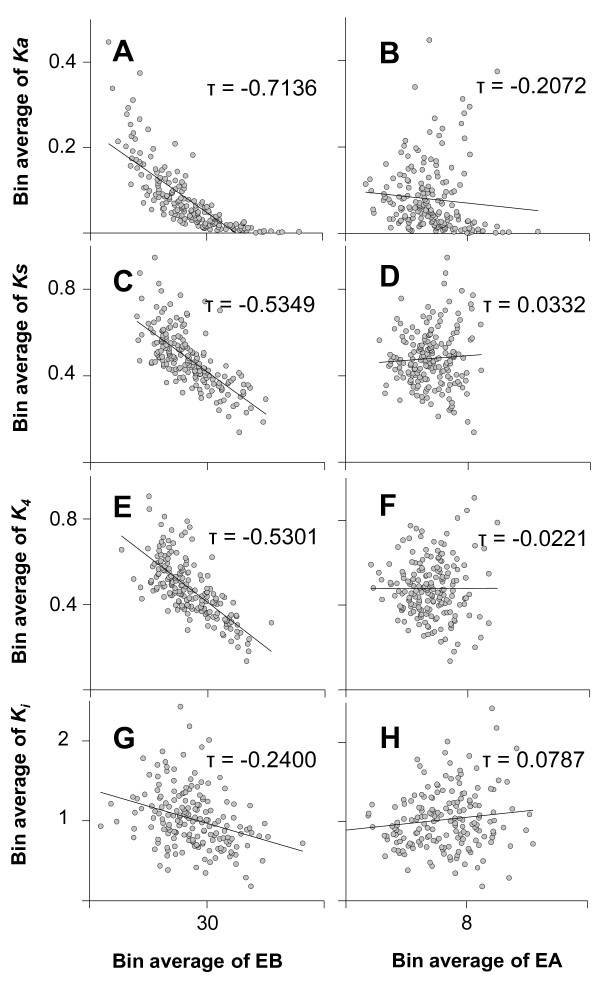
**Negative correlation between evolutionary rates and expression parameters**. All the data were grouped into 191 bins, with each bin containing 50 different genes. The data points are the averages of each bin. Each tau was generated from Kendall's rank correlation tests between EB and *Ka*, p = 2.35e-48 (**A**), between EA and *Ka*, p = 2.18e-05 (**B**), between EB and *Ks*, p = 6.36e-28 (**C**), and between EA and *Ks*, p = 0.49 (**D**). The lines in each graph were estimated by linear regression analysis. As described in the main text, the correlation between EB and rates (indicated by *Ka *or *Ks*) seems to be stronger than that between EA and rates (indicated by *Ka *or *Ks*). It is important to note that there is no statistical significance in the correlation between EA and *Ks*.

The evolutionary rates of genes, *Ka *and *Ks*, were plotted against EA and EB, respectively, to evaluate how gene expression parameters correlated with rates. Both expression parameters showed significant negative correlations with the rates by Kendall's rank correlation tests (Additional file 1, Figure S3A and S3B). We performed the same tests after the datasets were grouped into 191 bins of 50 genes each. Consistent with previous reports [7,18,22,23,30], EB showed significant negative correlations with the evolutionary rate, *Ka *(Figure 2A; Additional file 1, Figure S4A). In addition, EA showed negative correlations with *Ka *(Figure 2B; Additional file 1, Figure S4B). Notably, the negative correlation between EB and *Ka *(Kendall's tau = -0.7136, p = 2.35e-48) was much larger than that between EA and *Ka *(Kendall's tau = -0.2072, p = 2.18e-05), implying that the breadth rather than the level of gene expression might have more influence on determining the rates of evolution.

Several studies using unicellular organisms such as *S. cerevisiae *have shown that the expression levels of genes are highly correlated with synonymous substitution rates (*Ks*) because highly expressed proteins prefer optimal codons in the third codon position during translation elongation, resulting in codon usage bias [31-37]. In other words, the synonymous sequences of genes that are expressed at high levels are under stronger evolutionary constraints due to their requirements of optimal codon usage and therefore evolve more slowly than those of genes that are expressed at low levels. Interestingly, our data showed that EB was negatively correlated with *Ks *(Kendall's tau = -0.5349, p = 6.36e-28, Figure 2C; Additional file 1, Figure S4C), while EA did not show a significant negative correlation with *Ks *(Kendall's tau = 0.033, p = 0.49, Figure 2D; Additional file 1, Figure S4D). Next, we used *K*_*4*_, a measure of the evolutionary rate of four-fold degenerate site (Figure 2E and 2F), and *Ki*, a measure of intron evolutionary rate, for further analysis (Figure 2G and 2H). Interestingly, the negative correlations between EB and *K*_*4 *_(Kendall's tau = -0.5301, p = 1.91e-27, Figure 2E), and EB and *Ki *(Kendall's tau = -0.2400, p = 1.88e-06, Figure 2G) became weaker, while the negative correlations disappeared between EA and *K*_*4 *_(Kendall's tau = -0.0221, p = 0.65, Figure 2F), and between EA and *Ki *(Kendall's tau = 0.0787, p = 0.11, Figure 2H). These results suggest again that the breadth rather than the level of expression is an important component in determining the evolutionary rates of genes in multicellular organisms.

### Random shuffling of EB or EA for each gene shows that the negative correlations of EB with the rates are significant

**Figure 3:**
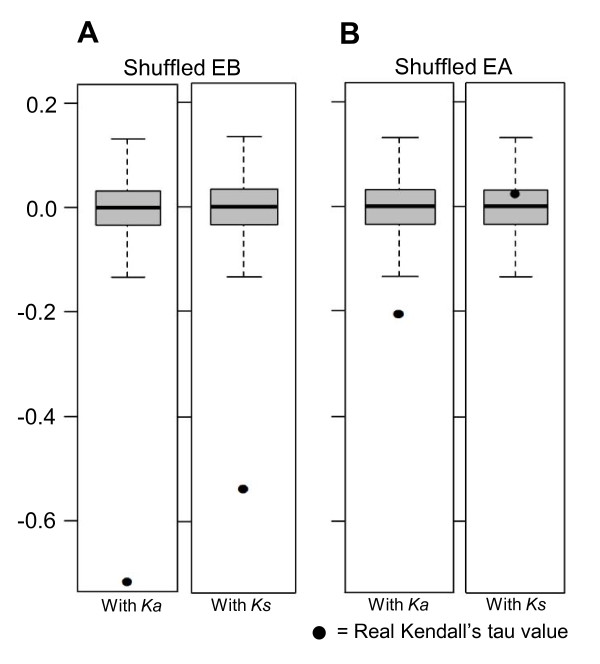
**Effect of random shuffling analysis of EA and EB on the correlation with evolutionary rates**. These graphs show the box plot analysis of the tau values generated by Kendall's correlation tests during 10,000 random shuffling experiments involving EB and EA values among genes comparing the original tau (indicated by black solid dots) between EB and rates (**A**) and between EA and rates (**B**). Details can be found in the Methods section.

We performed a random shuffling analysis of EAs and EBs to determine whether the correlation coefficient values (Kendall's tau) derived from Kendall's tau correlation tests between the expression variables and rates were significantly different from those expected by random chance. Briefly, the original EB and EA values were randomly shuffled among genes, Kendall's correlation tests were performed for each randomized shuffling event, and a tau value was obtained for each run. The shuffling experiments and correlation tests were executed for 10,000 iterations, and the tau values from the real data were compared with those of randomized shuffling to determine the deviation of tau from chance. Figure 3A clearly shows that the correlation coefficient values observed in the real data could not have been generated by random shuffling (p < < 0.00001), meaning that the negative correlations between the EBs and the rates (*Ka*, or *Ks*) are statistically significant (Figure 3A). The same analysis against the correlation of the EAs and rates revealed that the negative correlation between EA and *Ks *is not significant, but the negative correlation between EA and *Ka *is statistically significant (Figure 3B). This test supported our hypothesis that the breadth of expression has a greater impact on the rate of evolution than the abundance. The tau for the EB and *Ka *correlation was located much farther from the lowest quantile of the randomized shuffling than the EA and *Ka *correlation. As shown in Figure 3, EB has a stronger negative correlation with *Ks *than EA does with *Ks*, suggesting that even the synonymous substitution rate was more strongly correlated with EB than with EA.

### The negative correlations between EAs and evolutionary rates disappear when the EBs are even

Because EA is correlated with EB in multicellular organisms, it is difficult to analyze the two variables separately in terms of their relationship to evolutionary rates. Several previous studies have already shown the same negative correlation between the two values and the evolutionary rates [18,19,22,23,27], but they did not discriminate between the individual influence of EA and EB on the rates. In multicellular organisms, the breadth of gene expression is also critical for cell differentiation and development, similar to the level of expression in unicellular organisms [27,38]. Therefore, one of the key purposes of our study was to distinguish differences between the two values and their correlations with evolutionary rates. To this end, we designed a novel approach, named 'fixed group analysis (FGA).'

**Figure 4:**
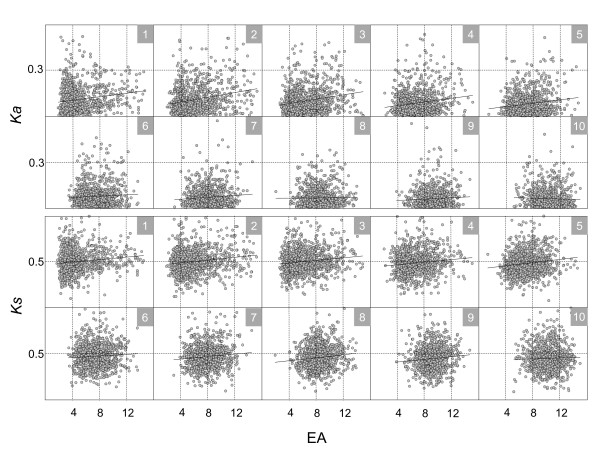
**Negative correlations between EAs and evolutionary rates disappear when EBs are constrained to be even**. The data from similar ranges of EBs were grouped together, resulting in 10 different groups. The genes in each group have similar EBs but different ranges of EAs and evolutionary rates. The boxes with nested numbers in the right upper corner (in grey) indicate the groups, and the numbers in the boxes correspond to those in Table S1A of Additional file 2. The lines in each group are derived from linear regression analysis data. All the groups consistently show that the negative correlations between EAs and evolutionary rates are reversed when the EBs are fixed as evenly as possible.

Briefly, the genes that showed similar breadth were grouped together. In this paper, all the gene pairs were divided into ten different groups. Each group included roughly the same number of genes (Additional file 2, Table S1A and B) and a similar range of EB values. We expected that this FGA approach would minimize the effect of unwanted contributions from EBs to the correlations between EAs and evolutionary rates, and vice versa. Then, the correlations between EAs and evolutionary rates were estimated for each group to see if the negative correlation pattern was maintained even after the effects of EBs on the rates were controlled as evenly as possible. As shown in Figure 4, the negative correlation between EA and *Ka *disappeared and instead was reversed when the EBs were even, such that nine out of ten groups showed positive correlations. The fixation of EB affected *Ks *in the same way as *Ka *(Figure 4; Additional file 1, Figure S5; Additional file 2, Table S1A and B).

### The negative correlations between EBs and evolutionary rates are obvious when the EAs are even

**Figure 5:**
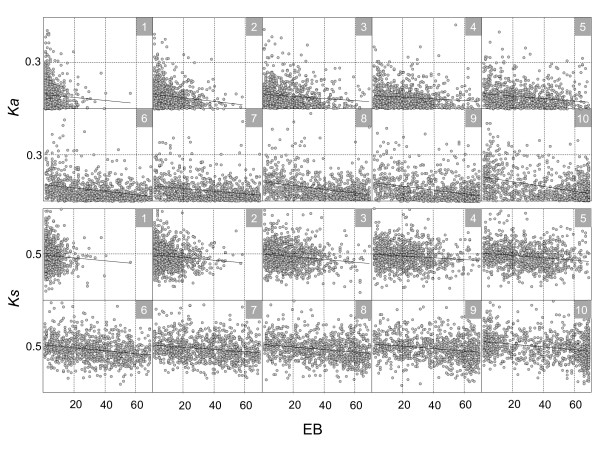
**Strong negative correlations between EBs and evolutionary rates are maintained when EAs are fixed**. Data with a similar range of EAs were grouped together, resulting in 10 different groups. The genes in each group have similar EAs but different ranges of EBs and evolutionary rates. The boxes with nested numbers in the upper right corner (grey) are the groups, and the numbers in the boxes correspond to those in Table S1A of Additional file 2. The lines in each group are derived from linear regression analysis results. All the groups consistently show that the negative correlations between EBs and evolutionary rates are maintained when the EAs are fixed as evenly as possible.

We applied the same FGA approach in an inverse way such that each EA was grouped as evenly as possible into ten different groups; then, the correlations between EBs and evolutionary rates were investigated in each FGA set. Interestingly, EBs maintained a strong negative correlation with the rates in all ten evenly grouped EAs and with statistically significant p-values (Figure 5; Additional file 1, Figure S6; Additional file 2, Table S1A and B). The FGA analyses were performed with several different grouping sizes of EA and EB values (data not shown), and a consistent trend emerged. The negative correlations were eliminated between EAs and the rates when the EBs were even, but were maintained in all ten groups between EBs and the rates when the EAs were even. All FGA groups except for group 1 showed this pattern with statistical significance, as shown in Table S1A of Additional file 2. These results consistently reflect that the breadth of gene expression has a bigger impact on rates than the level of gene expression.

### EBs are still negatively correlated with evolutionary rates when gene compactness or essentiality is controlled

**Figure 6:**
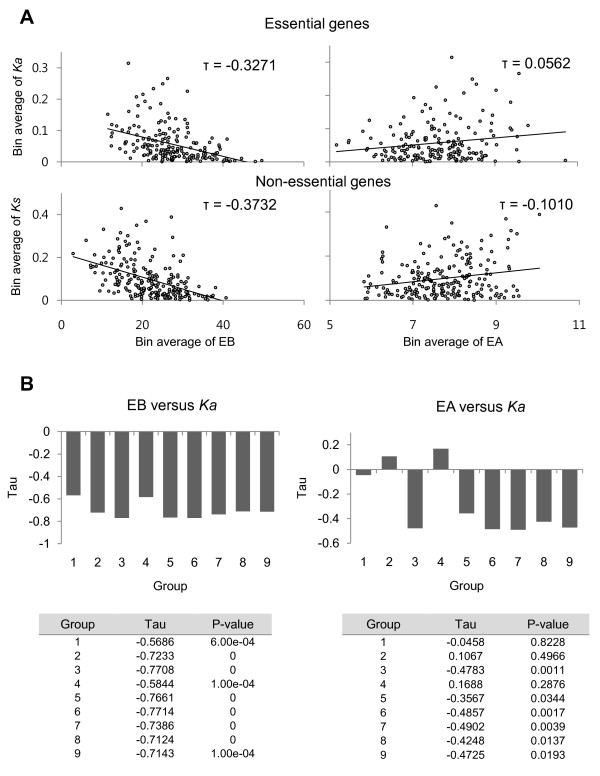
**The correlations between EBs and *Ka *when gene compactness or essentiality is controlled**. (**A**) Essentiality was estimated by mouse KO phenotypes (Methods). A total of 1,868 essential genes and 2,124 non-essential genes were identified. In each group, ten different genes were grouped together into a bin, and a total of 186 bins of essential genes and 212 bins of non-essential genes were made. Kendall's rank correlation tests were used to see the correlations between EBs and *Ka *in each group. **(B**) The information about gene structure was retrieved from UCSC (Methods). A total of 9,025 genes with all the information needed for this analysis, including intron number, EB, EA, and *Ka *were selected. Then all the genes were grouped into nine different groups, with each group containing a similar number of genes (717-1112). For each group, the correlation tests between EBs and *Ka *were performed using Kendall's rank correlation test. The tau values and p-values for the correlation tests in each group are shown as a box graph (top) and table (bottom), respectively.

Gene essentiality and gene compactness have been reported to be involved in determining evolutionary rates. We first investigated if the negative correlation of EBs remained when the essentiality of genes was controlled. The orthologous gene pairs between humans and mice were grouped into essential genes and non-essential genes by inferring the mouse KO phenotype data (Methods). Then the correlations between expression parameters and evolutionary rates were investigated in the essential gene and non-essential gene groups. As shown in Figure 6A, EBs were still negatively correlated with *Ka *in both groups of genes. In contrast, EAs showed a slightly positive correlation with *Ka *(Figure 6A).

Next, we investigated if the negative correlations of EBs with *Ka *were still maintained when the compactness of genes was controlled. The genes were grouped by the intron numbers of genes, such that the genes with a similar number of introns were grouped together. In each group, correlation tests were performed between EBs and *Ka*, and between EAs and *Ka*. As shown in Figure 6B, EBs were strongly negatively correlated with *Ka *in all of the gene groups, while the correlations between EAs and *Ka *showed a weak negative or even positive correlation in some groups (Figure 6B). This result confirmed that EBs are a more important determinant of evolutionary rate than EAs.

### The degree of negative correlation between EBs and evolutionary rates varies for different tissue types

**Figure 7:**
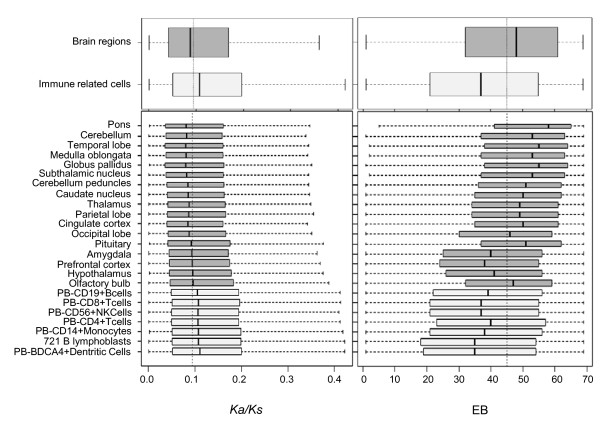
**A magnified view of the relationship between *Ka/Ks *and the EBs of genes expressed in brain- or immune-related tissues (or cells)**. The upper graphs show the box plots for *Ka/Ks *and EBs corresponding to all the brain-related genes and all the immune-related genes, as indicated. The lower graphs are magnified views of the box plots of the upper graphs, so the values from the genes expressed in each brain-related tissue and immune-related cells are shown individually.

The 'tissue-driven' hypothesis suggests that genes evolve at different rates according to the types of tissues where the genes are expressed. For example, genes expressed in brain-related tissues evolve most slowly [16]. Our results corroborate this hypothesis, as shown in Figure S7 of Additional file 1, as the rates of evolution vary among different tissue types. Specifically, the genes expressed in brain-related tissues, such as the amygdala, thalamus, and pons, evolve more slowly than other genes, while the genes expressed in immune-related cells evolve rapidly. Figure 7 is a magnified pattern of selected tissues of samples in Figure S7 of Additional file 1 (Figure 7). We selected 17 different brain-related tissues and 7 different immune-related cells to show the relationship between *Ka/Ks *and EBs. Interestingly, the more slowly evolving brain-related genes have wider expression while the rapidly evolving immune-related genes have a narrower expression pattern. Strangely, genes expressed in the liver or lung did not fit this trend (Additional file 1, Figure S7), as they were more widely expressed than genes expressed in other tissues, yet they evolved rapidly. While we have no obvious explanation for this trend, tissue-specific evolutionary constraints might influence the evolutionary rates of genes. Overall, the expression breadth consistently has a bigger impact on the evolutionary rates than the expression level.

## Conclusions

Recent research using genome-scale data of sequences, mutants, and PPIs has revealed that several genomic parameters such as expression breadth, expression abundance, PPI, and essentiality exhibit statistically significant correlations with evolutionary rates [9-12]. Several studies have argued that expression abundance is the most important genomic parameters, for correlation with protein evolutionary rate [12-15]. Considering that expression breadth is another dimension of gene expression in addition to expression abundance in multicellular organism, we investigated the influence of the two expression parameters, EA and EB, on the correlations with the rates of evolution. By employing a novel statistical method called 'FGA' and a random shuffling test, we showed that expression breadth is more closely related to evolutionary rates than expression abundance. We think that our study may contribute to a better understanding of what determines evolutionary rates of proteins in multicellular organisms.

The reason for the breadth of expression being more tightly correlated with the rate of evolution than the abundance of expression in multicellular organisms is not entirely clear. In fact, the two different measures, EA and EB, are not easily separable, as shown in Figure 1, meaning that genes with higher expression levels are more likely to be broadly expressed genes. Considering that the function of a tissue-specific gene is limited to specific tissue types, the evolutionary constraint influencing the rate of protein evolution should be weaker in tissue-specific genes than in the broadly expressed genes, thus explaining why the expression breadth of a gene is correlated with its rate of evolution.

Many studies have shown that expression breadth is the main determinant of the evolutionary rate of gene. For example, Tuller et al. (2008) reported that genes expressed in the cortical region, a more recent region of brain, evolve more slowly than those expressed in the subcortical region, a more ancient region of brain. They tried to explain this unexpected phenomenon using the 'preferential attachment' hypothesis suggested by Albert et al. (2002), in which genes expressed in the more recent cortical region are more likely to be expressed broadly because they tend to be the genes that already have a broad expression breadth in the subcortical region [27,39].

In the present paper, we also confirmed a similar pattern for the slow evolution of brain-related genes, namely that the brain genes evolve slowly because they have more broad expression patterns than genes expressed in other tissue types (Figure 7). The same scenario can also be applied to the evolution of immune-related genes (Figure 7), which are known to evolve rapidly [40-42]. The fast evolution of immune genes has been considered to be a signature of positive selection [40-43]. If immune-related gene evolution follows our hypothesis, the immune genes evolve rapidly because they are expressed in a narrow range of tissues.

Distinguishing the influence of EA and EB on evolutionary rates is useless when studying the evolutionary mechanisms of genes in unicellular organisms. However, since the breadth and abundance have different roles in cell differentiation and organism development in multicellular organisms, it is reasonable to assume that the two variables have different influences on gene evolution.

The compactness of genes, i.e., the length or the number of introns, could also influence gene evolution [24,29,38,44,45]. Several recent papers have reported contradictory findings on the relationship between expression parameters and the lengths of introns [44,46,47]. Some argue that highly expressed genes are more compact to reduce the cost of transcription ('selection for economy' model), while others think that narrowly expressed genes are not compact because the introns or noncoding regions of the genes are involved in more complex expression regulation ('genome design' model). Some papers have reported contradictory findings that housekeeping genes are not as compact as expected [48]. The correlation between gene compactness and gene expression parameters is not independent of other correlations. Therefore, the relationship between compactness and expression should also be determined when considering the correlation between expression parameters and evolutionary rates.

We hope that these efforts will significantly contribute toward questions related to genome evolution in the future.

## Methods

### Microarray data

The GDS596 dataset derived from the U133A Affymetrix chip was downloaded from (ftp://ftp.ncbi.nih.gov/pub/geo/DATA/supplementary/series/GSE1133/GSE1133_RAW.tar) [49]. A normalization procedure was performed for the 158 raw CEL files from the GDS596 dataset generated from the Affymetrix chip U133A (Santa Clara, CA) using the gcRMA method [50] incorporated in Bioconductor (Linux version 2.9.1) [51]. Unlike other studies using an arbitrary cutoff, such as 200, 250, or 300, we applied three different methods for AP calls: the Affymetrix MAS5 AP call method [52], the MAS5 AP calls based on GC-RMA transformed PM threshold values [53], and the PANP method [54]. The resulting output using the second method is presented herein. When one of the replicates was present, we recorded a presence variable. The probes labeled as '_x_at' or '_s_at', which represented a higher likelihood of cross-hybridization, were removed from the dataset, and the intensity of a gene was subsequently estimated as the average of the remaining probes. In the case where all the probes for a specific gene were represented by '_x_at' or '_s_at', the average of those probes was calculated. Of a total of 79 tissues, only 69 were chosen for our analyses after removing the tissues from the 'disease' or 'fetal' classes. The datasets generated from the different methods led to essentially the same conclusions as described in the main text (Additional file 1, Figure S7).

### EST collection and selection

The EST data sets were downloaded as the files Hs.lib.info.gz and Hs.data.gz from the UniGene database (ftp://ftp.ncbi.nih.gov/repository/UniGene/Homo_sapiens/) with the latest version on 2009-10-29. The Hs.data file contains 123,396 EST clusters, and the Hs.lib.info file has 8,681 cDNA libraries. We decided to use only normal adult tissues for the analyses, and the ESTs derived from cDNA libraries of disease or fetal tissues were excluded from our analyses using the keyword search. Briefly, after the cDNA library information of Hs.lib.info was sorted by gene IDs, a total of 3,675 cDNA libraries containing 'Normal' tissues as delimited by the "CANCER_SOURCE" tag were selected. Next, 2,310 cDNA libraries out of the 3,675 were chosen based on information delimited by using the 'DEVELOPMENTAL_STAGE' tags of adult and juvenile. Furthermore, considering that the tissue information was not deposited systematically in the data file, we manually inspected the information as delimited by TITLE, TISSUE, VERBATIUM_TISSUE, and CELL_LINE_SOURCE, and 2,105 cDNA libraries were finally selected. These 2,105 cDNA libraries were then subdivided into different tissue types. From the Hs.data containing UniGene clusters, Entrez IDs were extracted and used to compare gene lists for evolutionary information. Combining all the cDNA library, EST, and gene ID information, the number of ESTs for each different type of tissue was estimated. The tissues containing fewer than 3,000 ESTs per tissue and the tissues with no exact definition, as indicated by 'mixed' and 'uncharacterized tissue,' were removed from the analyses. After performing these cleaning steps, a total of 36 different tissues containing 507,140 ESTs remained. From these ESTs, the EAs were estimated by LOG_2 _transformation of the proportion of ESTs corresponding to a given gene. The EB was defined as the sum of the types of tissues that a given gene expresses at or above a threshold (we tested the threshold from 1 to 5, and the conclusions from the analyses were similar).

### Collecting mouse knock-out data and gene compactness data

The HMD_HumanPhenotype.rpt file containing a total of 5,851 genes having MP IDs and the Mpheno_OBO.ontology file with definitions corresponding to MP IDs were downloaded from the ftp site, ftp://ftp.informatics.jax.org/pub/reports/index.html of the MGI site (http://www.informatics.jax.org/) on Jan 5, 2010. A total of 3,992 genes that have EB, EA values and ortholog information between human and mouse were used for this analysis. Following the definitions nested in the Mpheno_OBO.ontology file, the genes were grouped into essential and non-essential genes. Next, using the "Table browser" of the UCSC site (http://genome.ucsc.edu), information about the genes and gene structures for GRCh37/hg19 genome assembly, such as location, length, and number of exons and introns, was obtained.

### Calculating the rates of evolution

To calculate the evolutionary rates of genes corresponding to the transcripts chosen for our analysis above, we downloaded the human and mouse RefSeq cDNA sequences from ftp://ftp.ncbi.nih.gov/refseq/, identified ortholog pairs using the HomoloGene data (ftp://ftp.ncbi.nih.gov/pub/HomoloGene/current/homologene.data), and linked them to the Unigene transcripts. Only the curated sequences with NM prefixes were chosen. The BLASTP program was used to search the best hits, and a total of 17,629 ortholog candidates were identified. The coding sequences of the two species were aligned with ClustalW. The evolutionary rates *Ka*, *Ks*, *Ka/Ks*, and *K*_*4 *_were calculated using Li's method [55]. For the gene pairs with *Ks *values that were too high, 0<*Ks *<1 was excluded to reduce statistical noise for our analysis. *Ki *values were estimated by the method described by Gazave et al. (2005) using aligned human and chimpanzee introns [56].

### Fixed group analysis (FGA)

To discriminate the effects of EA and EB on the rates of evolution, we designed a novel approach named the 'fixed group analysis (FGA)'. Briefly, the genes showing a series of similar breadths are grouped together in the same group. In this paper, all the gene pairs were divided into 10 different groups. Each group included roughly the same number of genes (877-1,038) with a similar EA or EB, as shown in Table S1 of Additional file 2. The correlation coefficients using Kendall's tau correlation tests were estimated for each group to see if the negative correlation pattern was maintained even after the effects of breadth or abundance on the rates were fixed.

## Authors' contributions

SSC conceived of the study, and drafted the manuscript. SGP wrote the program code and carried out the statistical analysis. All authors read and approved the final manuscript.

## Supplementary Material

Additional file 1 **Supplementary Figures**. Supplementary figures and information Click here for file

Additional file 2 **Supplementary Tables**. Supplementary tables Click here for file
