# Supplementary material for: Expression breadth and expression abundance behave differently in correlations with evolutionary rates
Source: BMC Evol Biol. 2010 Aug 7;10:241. doi: 10.1186/1471-2148-10-241 (PMC2924872; doi:10.1186/1471-2148-10-241)
Supplement: Additional file 1 — Supplementary Figures. Supplementary figures and information [file 1471-2148-10-241-S1.DOC]

**Supplementary Figures**


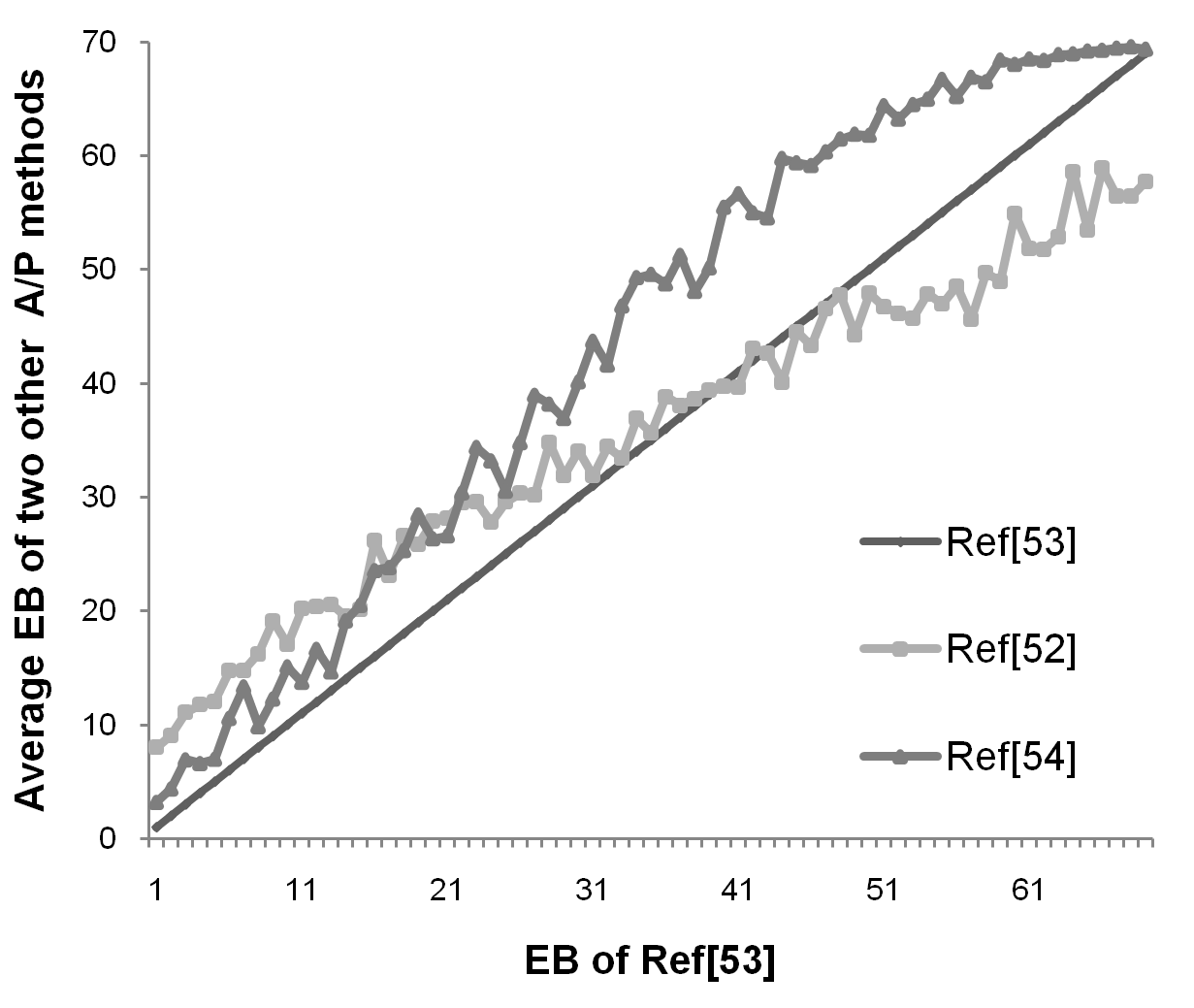


**Figure S1. Comparison of EB values generated by three different A/P call methods**

As described in the maintext, we tested three different AP call methods suggested by three different papers. We finally chose the method described by Schuster et al. (2007) [53]. Using the EB values we used as a standard, we plotted the EB values generated by the two other methods, Hubbell et al. (2002) [52] and Warren et al. (2007) [54], and found that the three different AP call methods were consistent overall.


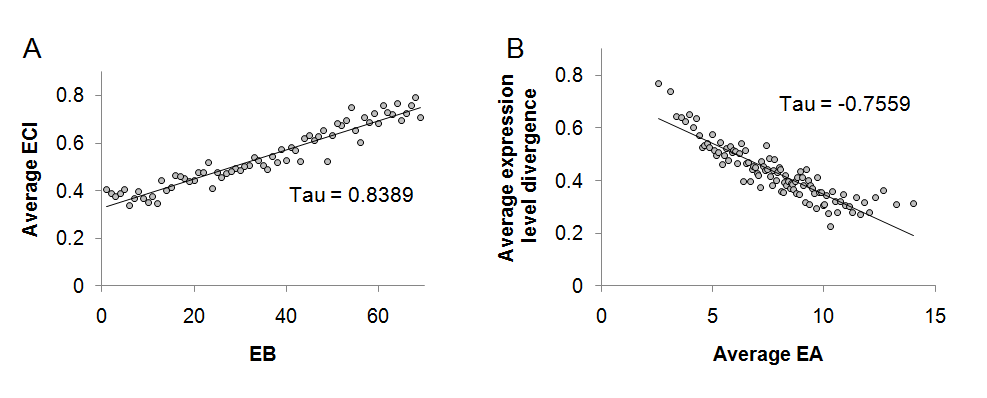


**Figure S2. Strong correlation between expression conservation index (ECI) and EB, and between expression level divergence (ELD) and EA**

The ECI values were estimated by Yang et al. (2005)’ method [18] while ELD values were estimated by Jordan et al. (2005)’s method [30]. A total of 5151 orthologous pairs between human and mouse were used for this analysis. All the genes were grouped into 103 different bins, 50 different genes were contained in each bin. **(A)** Positive correlation between ECI and EB, **(B)** Negative correlation between ELD and EA.


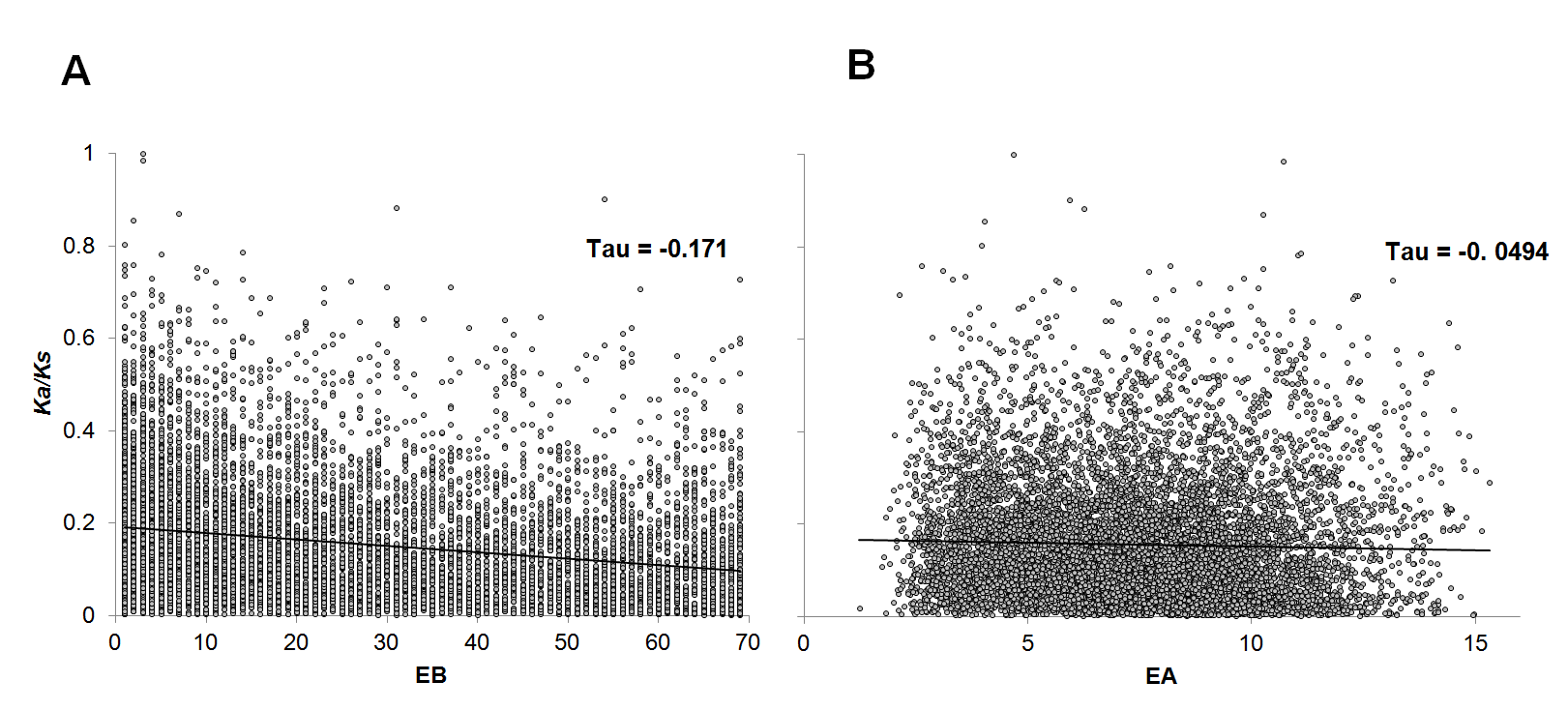


**Figure S3. The correlation between evolutionary rates (Ka/Ks) and expression parameters (EA and EB).**

A total of 9506 genes were selected from the microarray expression data derived from the GDS596 (see Materials and methods), and plotted against EB **(A)** and EA **(B)**. P-values obtained from the Kendall’s correlation tests were **(A)** 2.2e-135 and **(B)** 4.9e-13 respectively. These graphs consistently show that the expression parameters are correlated negatively with the evolutionary rates.


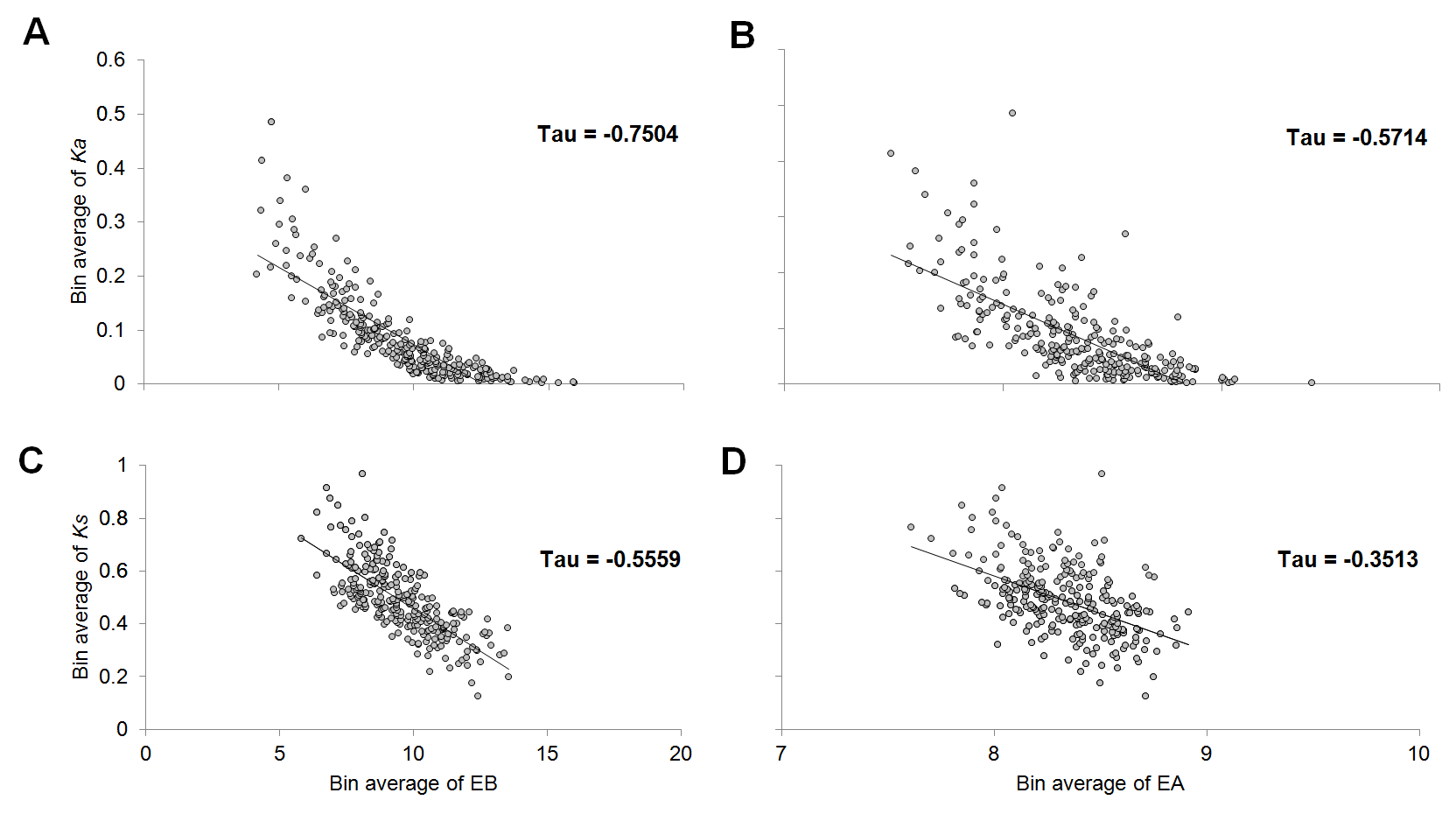


**Figure S4**. **Negative correlation between evolutionary rates and expression parameters in the EST dataset**

All the data were grouped into 272 bins, with each bin contains 50 different genes. The data points are the averages of each bin. Each tau was generated from the Kendall’s rank correlation tests between EB and *Ka*; p = 7.88e-76 **(A)**, between EA and *Ka*; p = 8.20e-45 **(B)**, between EB and *Ks*, p = 2.22e-42 **(C)**, and between EA and *Ks*; p = 5.91e-18 **(D)**. The lines in each graph were estimated by linear regression analysis. As described in the main text, the correlation between EBs and rates (indicated by *Ka*, or *Ks*) seems to be stronger than that between EAs and rates (indicated *by Ka, or Ks*).


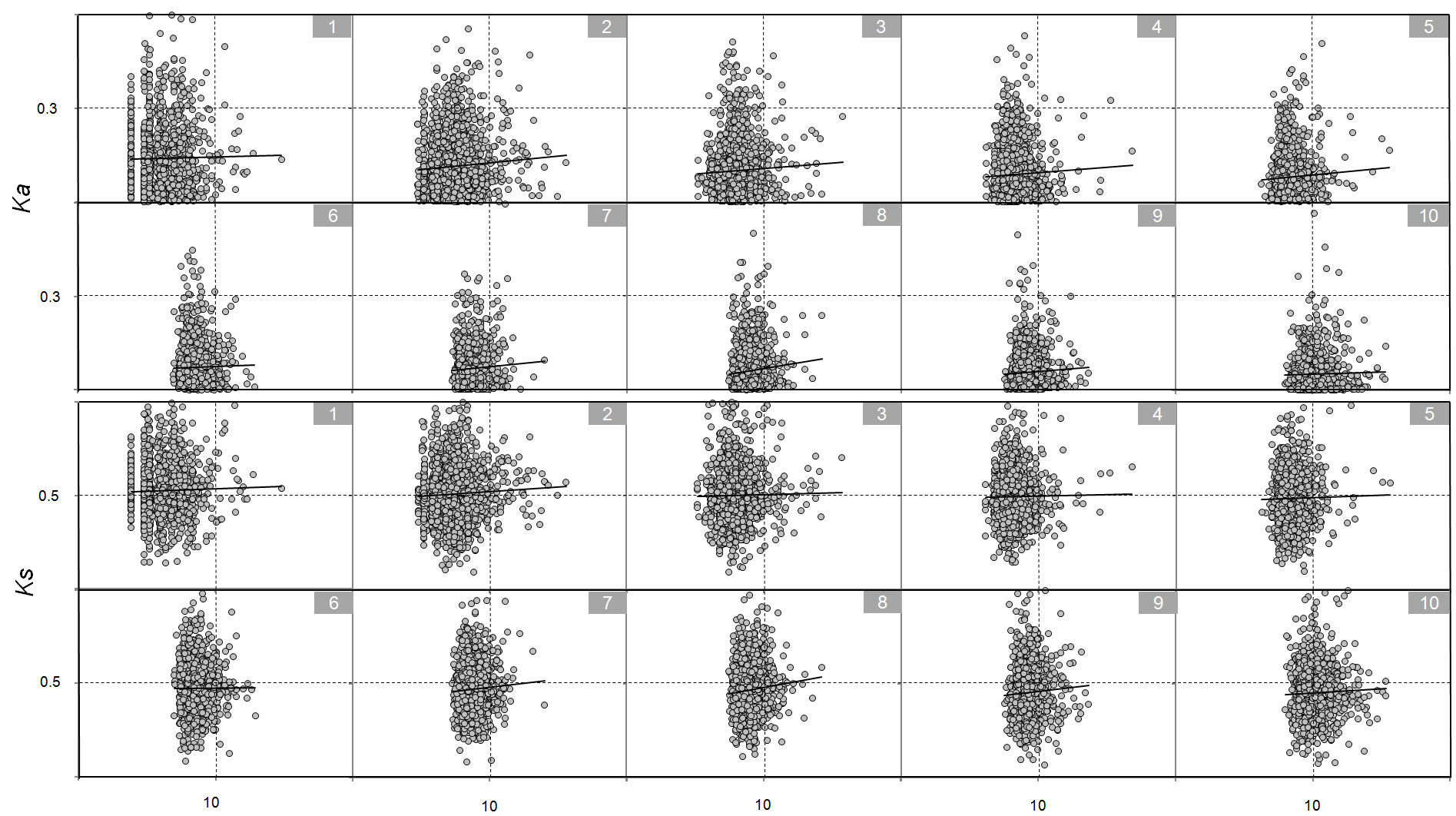


**Figure S5. Negative correlations between EAs and evolutionary rates disappear when EBs are constrained to be even in the EST dataset.**

The data from similar ranges of EBs were grouped together, resulting in ten different groups. The genes in each group have similar EBs but also different ranges of EAs and evolutionary rates. The boxes with nested numbers in the right upper corner (in grey) indicate the groups, and the numbers in the boxes correspond to those in Table S1A of Additional file2. The lines in each group are derived from linear regression analysis data. All the groups consistently show that the negative correlations between EAs and evolutionary rates are reversed when EBs are fixed as evenly as possible.


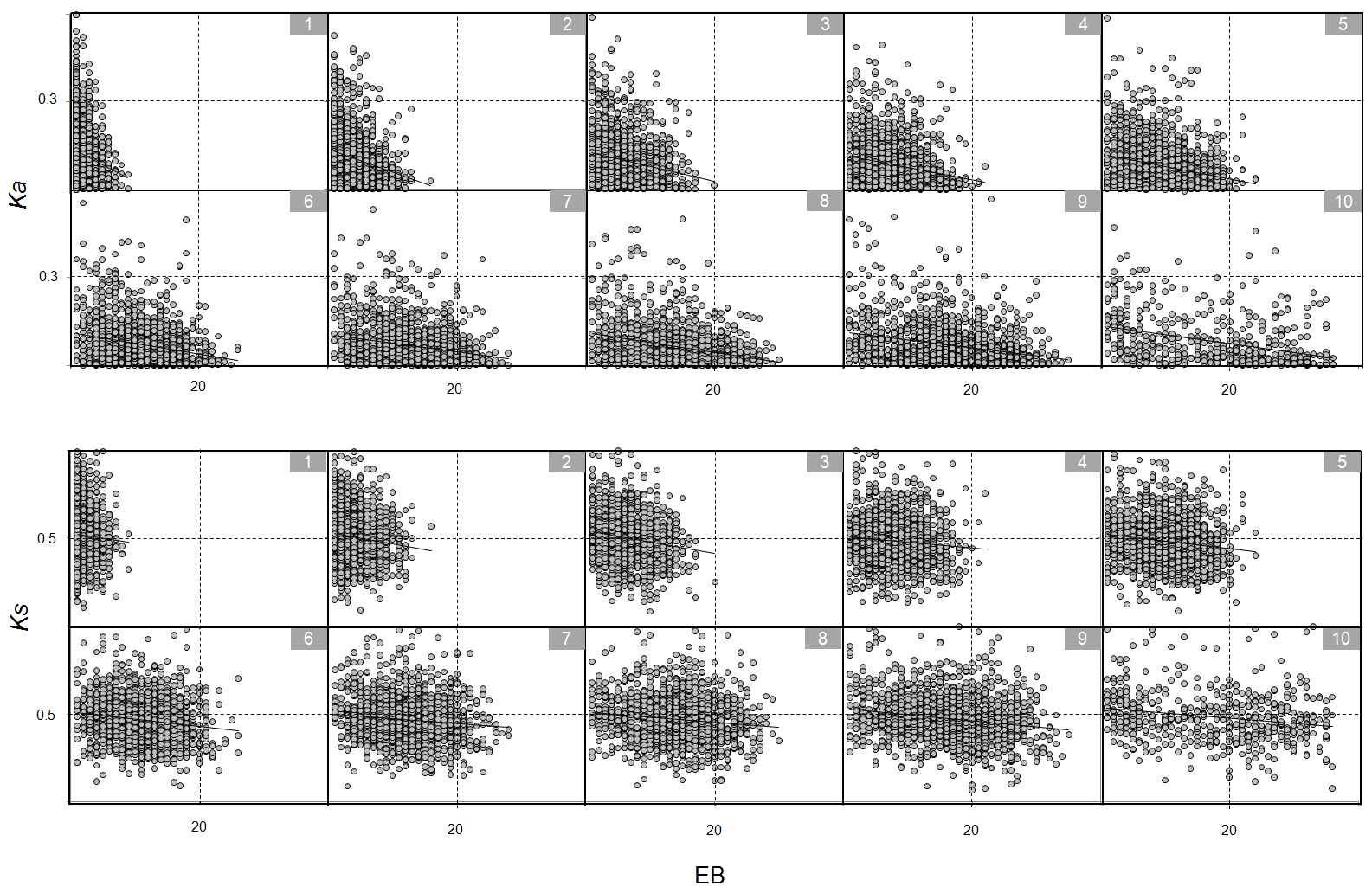


**Figure S6.** **Strong negative correlation between EBs and evolutionary rates are maintained when EAs are fixed as even in the EST dataset**

Data with a similar range of EAs were grouped together, resulting in ten different groups. The genes in each group have similar EAs but different ranges of EBs and evolutionary rates. The boxes with nested numbers in the upper right corner (grey) are the groups, and the numbers in the boxes correspond to those in Table S1A of Additional file 2. The lines in each group are derived from linear regression analysis results. All the groups consistently show that the negative correlations between EBs and evolutionary rates are maintained when EAs are fixed as evenly as possible.


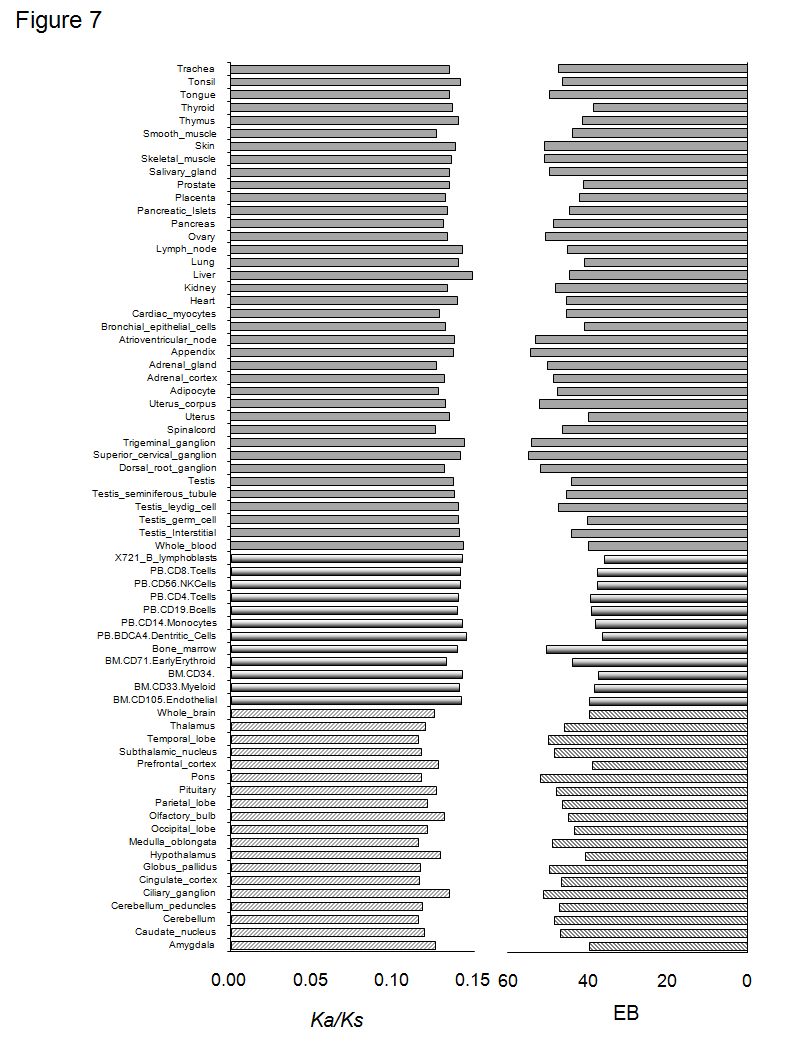


**Figure S7. The relationship between evolutionary rates and EB for different tissue types.**

The left graph indicates the average of *Ka/Ks*, as estimated by the genes expressed in different tissue types, while the right graph shows the average of the EBs generated by the genes. The bars with sloped lines are the values estimated from brain-related tissue, and the filled bars with gradients are derived from immune-related tissues. The grey filled boxes on the upper side of the graph indicate the values of the remaining tissues. This graph suggests that the evolutionary rates of genes expressed in each tissue are related to the extent to which genes are expressed.
