# Supplementary material for: Expression breadth and expression abundance behave differently in correlations with evolutionary rates
Source: BMC Evol Biol. 2010 Aug 7;10:241. doi: 10.1186/1471-2148-10-241 (PMC2924872; doi:10.1186/1471-2148-10-241)
Supplement: Additional file 2 — Supplementary Tables. Supplementary tables [file 1471-2148-10-241-S2.DOC]

**Table S1**

1. **Summary of the FGA analysis using the microarray dataset**

| **Group** | **Fixed EB group** | | | | | | **Fixed EA group** | | | | | |
| --- | --- | --- | --- | --- | --- | --- | --- | --- | --- | --- | --- | --- |
|  | **#Genes** | **R/EB** | ***Ka* versus EA** | | ***Ks* versus EA** | | **#Genes** | **R/EA** | ***Ka* versus EB** | | ***Ks* versus EB** | |
|  |  |  | **Tau** | **p-value** | **Tau** | **p-value** |  |  | **Tau** | **p-value** | **Tau** | **p-value** |
| 1 | 964 | 1~2 | 0.1200 | 2.46e-08 | 0.1078 | 5.50e-07 | 951 | 1.23~3.92 | -0.0442 | 5.65e-02 | -0.0158 | 4.94e-01 |
| 2 | 954 | 3~5 | 0.1452 | 1.92e-11 | 0.0847 | 9.04e-05 | 951 | 3.92~4.96 | -0.1074 | 1.24e-06 | -0.0962 | 1.40e-05 |
| 3 | 1038 | 6~10 | 0.1131 | 4.90e-08 | 0.0944 | 5.34e-06 | 951 | 4.96~5.80 | -0.1016 | 3.68e-06 | -0.1001 | 5.11e-06 |
| 4 | 1031 | 11~16 | 0.0770 | 2.17e-04 | 0.0781 | 1.75e-04 | 951 | 5.80~6.64 | -0.1060 | 1.29e-06 | -0.0880 | 5.78e-05 |
| 5 | 913 | 17~22 | 0.0544 | 1.39e-02 | 0.0976 | 1.02e-05 | 951 | 6.64~7.41 | -0.1557 | 1.02e-12 | -0.1338 | 9.08e-10 |
| 6 | 960 | 23~30 | 0.0043 | 8.44e-01 | 0.0382 | 7.69e-02 | 951 | 7.41~8.11 | -0.1866 | 1.23e-17 | -0.1681 | 1.34e-14 |
| 7 | 911 | 31~39 | 0.0330 | 1.36e-01 | 0.0602 | 6.57e-03 | 951 | 8.11~8.82 | -0.1738 | 1.59e-15 | -0.1267 | 6.27e-09 |
| 8 | 952 | 40~50 | 0.0010 | 9.62e-01 | 0.0735 | 6.85e-04 | 951 | 8.82~9.64 | -0.2457 | 2.06e-29 | -0.1599 | 2.32e-13 |
| 9 | 916 | 51~61 | 0.0322 | 1.45e-01 | 0.0680 | 2.06e-03 | 951 | 9.64~10.78 | -0.2480 | 6.86e-30 | -0.1538 | 1.88e-12 |
| 10 | 877 | 62~69 | -0.0596 | 8.29e-03 | 0.0143 | 5.26e-01 | 947 | 10.78~15.31 | -0.3040 | 1.86e-43 | -0.1754 | 1.54e-15 |

#, number of; R/EB, range of EB values in each group;R/EA, range of EA values in each group

1. **Summary of the FGA analysis using the EST dataset**

| **Group** | **Fixed EB group** | | | | | | **Fixed EA group** | | | | | |
| --- | --- | --- | --- | --- | --- | --- | --- | --- | --- | --- | --- | --- |
|  | **#Genes** | **R/ EB** | ***Ka* versus EA** | | ***Ks* versus EA** | | **#Genes** | **R/EA** | ***Ka* versus EB** | | ***Ks* versus EB** | |
|  |  |  | **Tau** | **p-value** | **Tau** | **p-value** |  |  | **Tau** | **p-value** | **Tau** | **p-value** |
| 1 | 1124 | 1 | 0.0134 | 5.06e-01 | 0.0161 | 4.24e-01 | 1461 | 3.84~6.81 | -0.1056 | 8.30e-08 | -0.0400 | 4.24e-02 |
| 2 | 1857 | 2~3 | 0.0266 | 8.76e-02 | 0.0316 | 4.22e-02 | 1419 | 6.81~7.44 | -0.1645 | 1.64e-18 | -0.0915 | 1.04e-06 |
| 3 | 1671 | 4~5 | -0.0019 | 9.10e-01 | -0.0126 | 4.44e-01 | 1449 | 7.48~7.79 | -0.1410 | 1.13e-14 | -0.1072 | 4.30e-09 |
| 4 | 1460 | 6~7 | 0.0020 | 9.07e-01 | 0.0005 | 9.77e-01 | 1511 | 7.80~8.09 | -0.1541 | 4.05e-18 | -0.0755 | 2.14e-05 |
| 5 | 1449 | 8~9 | -0.0188 | 2.87e-01 | -0.0113 | 5.20e-01 | 1475 | 8.12~8.43 | -0.1916 | 8.92e-27 | -0.1063 | 2.77e-09 |
| 6 | 1323 | 10~11 | 0.0071 | 6.98e-01 | -0.0044 | 8.13e-01 | 1537 | 8.43~8.74 | -0.1753 | 1.32e-23 | -0.1122 | 1.46e-10 |
| 7 | 1201 | 12~13 | 0.0024 | 9.01e-01 | 0.0228 | 2.39e-01 | 1427 | 8.74~9.07 | -0.1597 | 1.13e-18 | -0.0801 | 9.78e-06 |
| 8 | 1349 | 14~16 | 0.0624 | 6.25e-04 | 0.0546 | 2.77e-03 | 1402 | 9.07~9.59 | -0.1952 | 8.78e-27 | -0.0992 | 5.22e-08 |
| 9 | 1176 | 17~20 | 0.0466 | 1.70e-02 | 0.0612 | 1.74e-03 | 1401 | 9.60~10.78 | -0.1920 | 3.94e-26 | -0.1198 | 4.21e-11 |
| 10 | 994 | 21~36 | -0.0302 | 1.55e-01 | 0.0352 | 9.74e-02 | 522 | 10.78~16.82 | -0.2983 | 9.83e-24 | -0.1475 | 6.79e-07 |

#, number of; R/EB, range of EB values in each group;R/EA, range of EA values in each group
